# Supplementary figures and images for: Scalable and flexible inference framework for stochastic dynamic single-cell models
Source: PLoS Comput Biol. 2022 May 19;18(5):e1010082. doi: 10.1371/journal.pcbi.1010082 (PMC9159578; doi:10.1371/journal.pcbi.1010082)

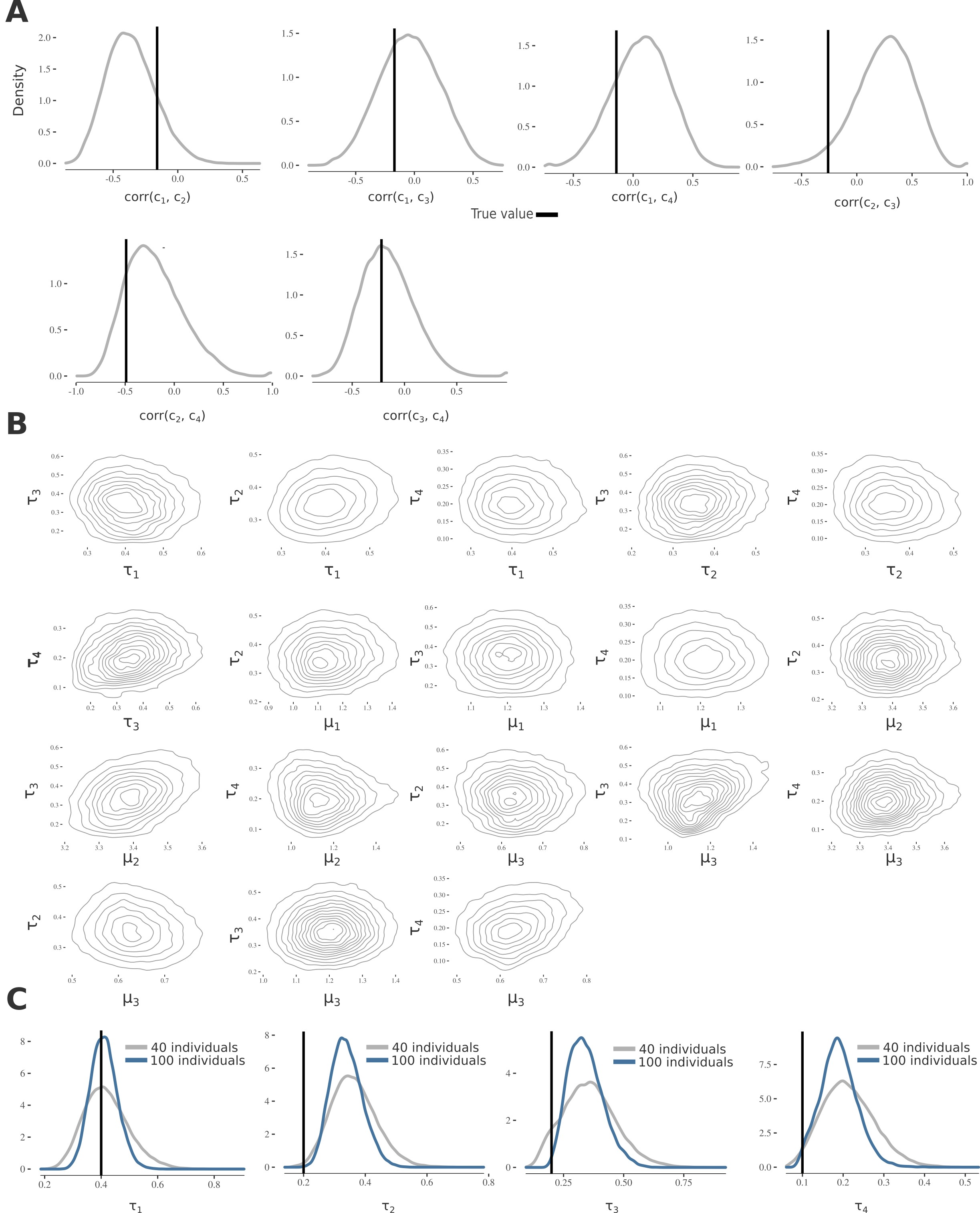

Supplement: S1 Fig — A) Marginal posterior from the inference run in Fig 2 for the correlation matrix Φ (non-diagonal values of the covariance matrix Ω). The correlation matrix characterises the correlation between the individual parameters (c1, c2, c3, c4). The black line represents the true-value. B) Pair plots for the three scale parameters that were problematic to infer, (τ1, τ2, τ3), against themselves and the log-mean values μi. C) Marginal posterior for the scale parameters (τ1, τ2, τ3, τ4) when simulating and doing inference for 40 (as in Fig 2) and 100 cells. Noticeably, albeit the parameter uncertainty decreases a bias still persist in (τ2, τ2, τ3) for the considered number of cells. However we notice that when using 100 cells, except for τ1 (where the difference is small), each posterior mode gets a little closer to the ground truth. (TIFF) [file pcbi.1010082.s001.tiff]

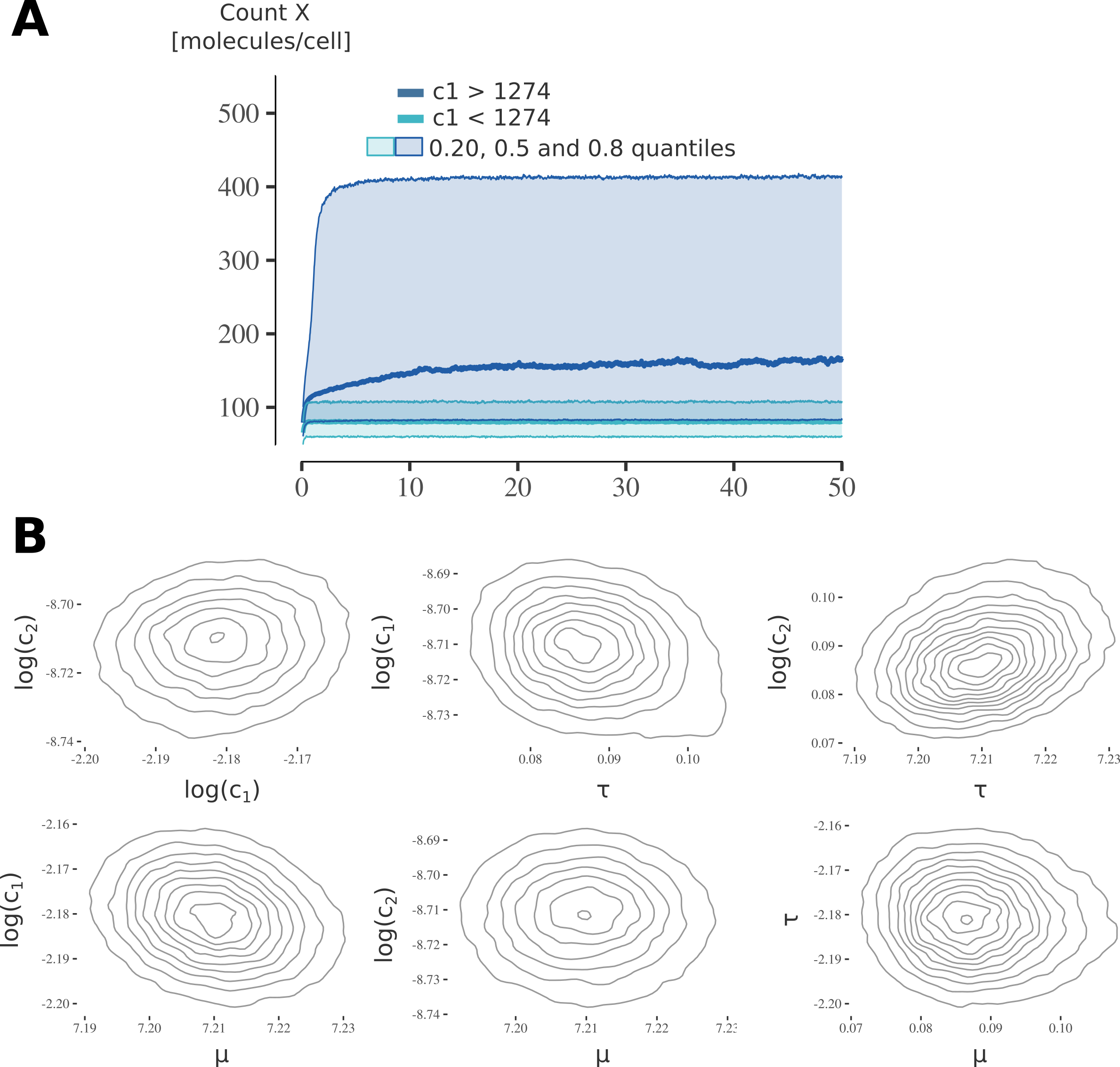

Supplement: S2 Fig — A) Using the inferred posterior for the Schlögl-model (Fig 3), 100, 000 cells were simulated. The cells were then split into the group having a synthesis rate c1 below 1274, and above 1274. For these groups the 0.2, 0.5 and 0.8 quantiles were computed. As seen from these quantiles, cells with a lower synthesis rate (c1) mainly commit to the lower cell-state (e.g low gene-expression). Meanwhile, cells with a larger synthesis rate commit to, and jump between, two different states of gene-expression. B) Marginal posterior pair plots for the inferred parameters. (TIFF) [file pcbi.1010082.s002.tiff]

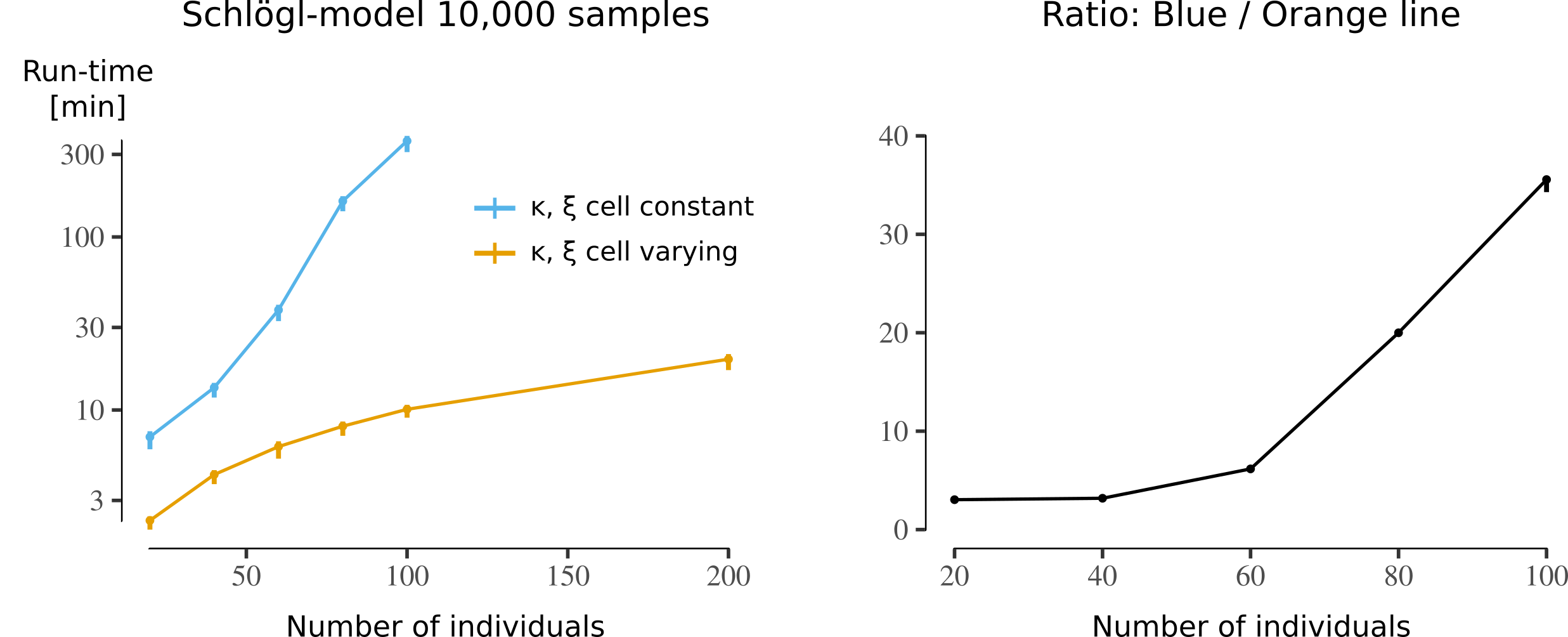

Supplement: S3 Fig — A) Comparison of run-time for the non-perturbed option (blue) where (κ, ξ) are constant between cells, and the default perturbed option (orange), where (κ, ξ) are slightly perturbed to vary between cells. Using the same-parameters as in Fig 3, data-sets for 20, 40, 60, 80, 100 and 200 individuals were simulated. Starting from true parameter values, the number of particles was tuned according to the tuning criteria (Section B in S1 Text) and PEPSDI was run for 10, 000 iterations. Same as for the inference in the Fig 3, we simulated intrinsic noise using the Langevin approximation, while guiding our particles using the modified diffusion bridge filter and correlating the particles with ρ = 0.999. For all data-sets, the particle tuning procedure suggested the use of 10 particles per individual for the perturbed-model option (orange-line), while for the non-perturbed option (blue line) the procedure suggested to use (with increasing number of individuals) 20, 20, 40, 130 and 230 particles for each individual (as described in S3 all individuals have the same number of particles for the non-perturbed sampler). The left plot shows the median run-time with max-and min values (bars) computed from three independent runs. Run-time was measured as the wall-clock time on a Dell Latitude with eight cores [Intel(R) Core(TM) i5–8365U CPU @ 1.60 GHz] running on Ubuntu 20.04. To minimise noise from other computer programs the benchmark was run on a freshly rebooted laptop with no other applications open. All the runs were performed sequentially using a single core. Due to the computational burden from performing all the runs sequentially, run-time was not measured for the case of 200 individuals for the non-perturbed option. B) Ratio between the blue and orange line, highlighting that the default perturbed option can be faster by more than a factor 30. (TIFF) [file pcbi.1010082.s003.tiff]

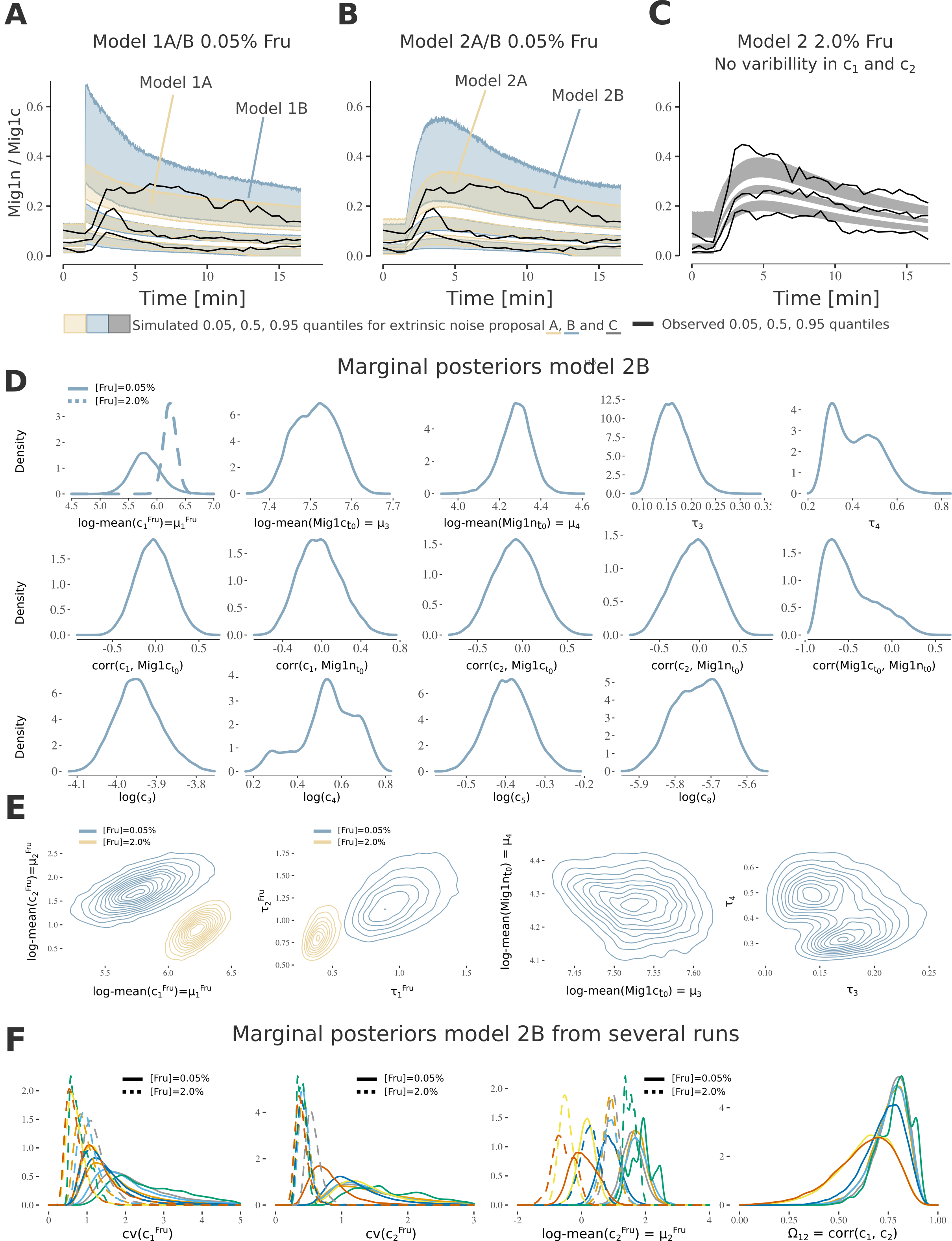

Supplement: S4 Fig — A-C) Posterior visual check for the 0.05% fructose data for model structure 1 and 2 using extrinsic noise-proposals A, B and (Fig 6). The credibility intervals were obtained as in Fig 2. Model 2B compared to model 2A has wider (but not biased) credibility intervals for the 0.05% fructose data, however, only model 2B accurately describes the 2% data (Fig 6F). C) Posterior visual check for model structure 2 and noise proposal A, where c1 and c2 do not vary between cells (no upstream extrinsic noise). Without upstream extrinsic noise the model fails to describe the observed cell-to-cell variability. Credibility intervals obtained as in A-B. D) Marginal posterior for the model-parameters not shown in Fig 6. E) Marginal pair posteriors for Model 2B for the the log-means and scale parameters (τ) for which the individual parameters were inferred to be correlated (corr(c1, c2) and corr(Mig1ct0, Mig1nt0) with colours representing fructose concentration. F) The same marginal posteriors as in Fig 6H from multiple inference runs (colors) with different starting values. The existence of several modes for μ2Fru (middle right) shows that the model is not fully identifiable (all parameters cannot be inferred unambiguously). However, some parameter relationships are consistent between runs. Namely, the coefficient of variation is larger in low fructose for (c1, c2) (two left plots), the log-mean μ2 is larger in low fructose (middle right), and the rates (c1, c2) are strongly correlated (right plot). (TIFF) [file pcbi.1010082.s004.tiff]

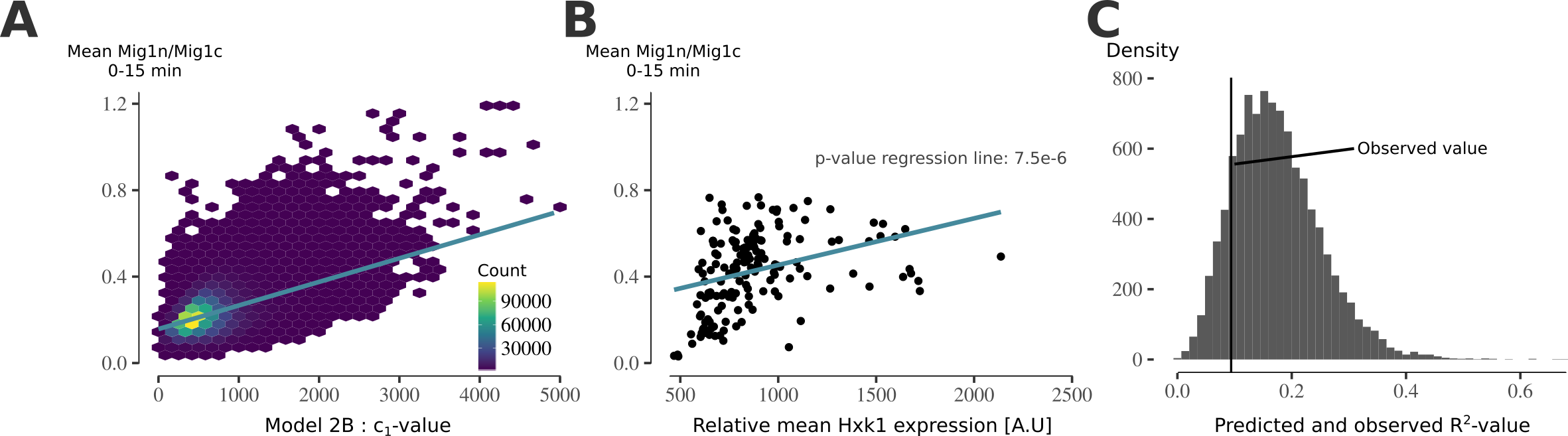

Supplement: S5 Fig — A) Mean Mig1 ratio over 15 minutes after 2% fructose addition versus the cell-varying model parameters c1 for model 2B obtained by simulating 1, 320, 000 cells. Noticeably, the cell-varying c1 of which hexokinase 1 (Hxk1) is a part (c1 ∝ [Hxk1]) explains a part of the cell-heterogeneity in Mig1 localisation. B) Mean Mig1 ratio over 15 minutes after 2% fructose addition versus relative mean Hxk1 expression for 132 cells obtained from single-cell time-lapse microscopy. The linear relationship is significant (p-value 7.5 × 10^−6^). The mean relative Hxk1 expression, which is likely proportional against Hxk1-expression, was computed by taking the mean of the Hxk1 expression over 240 min after fructose addition. C) Model predicted (bars) and observed (line) explained cellular heterogeneity (R2) in Mig1-localisation by relative Hxk1-expression and c1 respectively. The line is the R2-value (variability explained by regression line divided by total variability) for the linear regression in c), and the bars were computed by simulating mean Mig1 ratio (0–15 minutes after fructose addition) for 132 cells 10, 000 times, and computing the R2 for the Mig1n/Mig1c versus c1 linear regression for each instance. (TIFF) [file pcbi.1010082.s005.tiff]

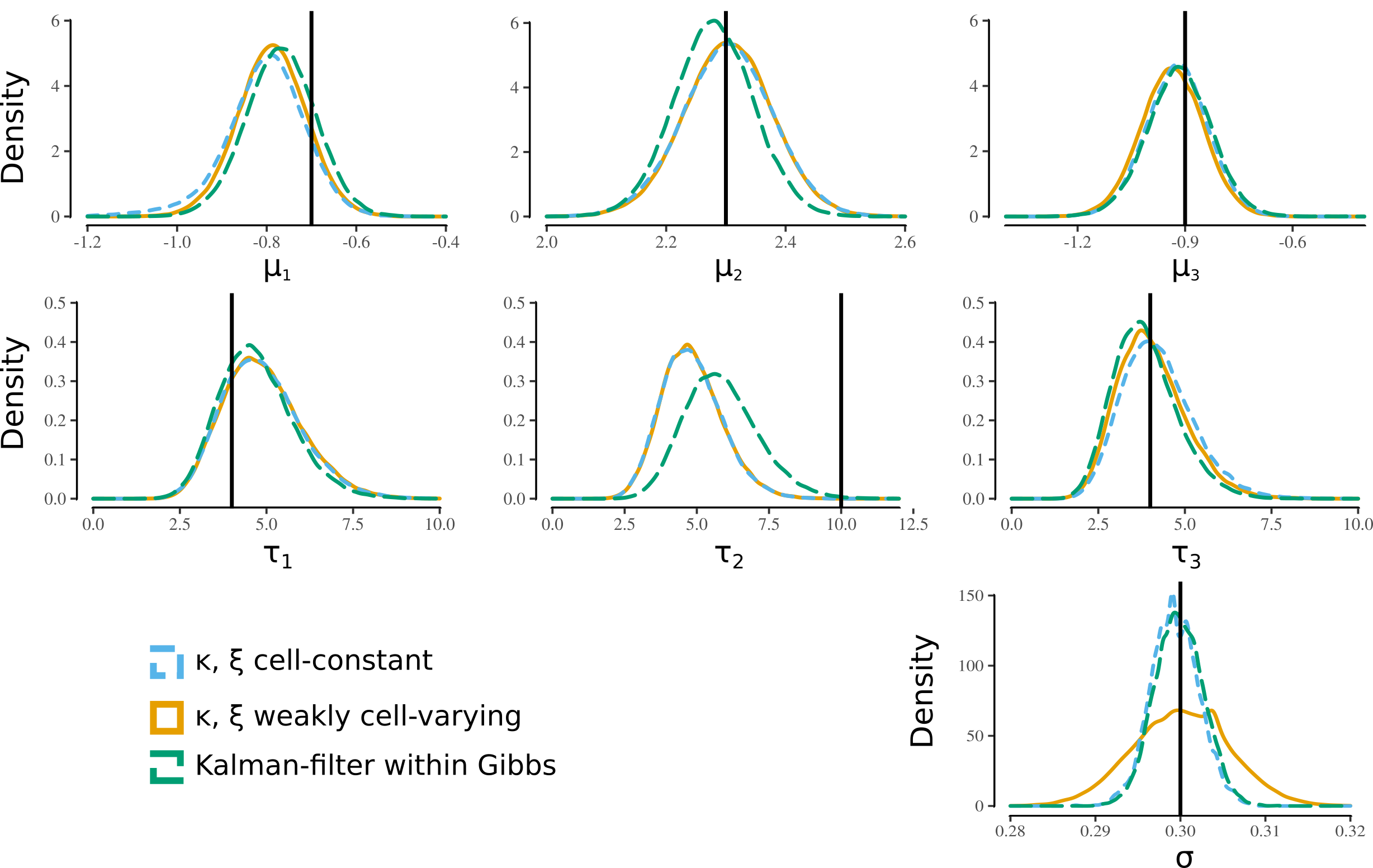

Supplement: S6 Fig — Inference was performed using PEPSDI with (κ, ξ) cell-constant and PEPSDI with (κ, ξ) weakly perturbed between cells (default option). These were compared against the gold-standard case, from Wiqvist et al. [10], where a Kalman-filter is embedded into the Gibbs-sampler (Alg 2 in S1 Text) for an exact evaluation of the likelihood. It can be seen that the consequence of perturbing the model is a slightly larger credibility interval for σ, however inference for the remaining parameters is remarkably similar to the non-perturbed case. (TIFF) [file pcbi.1010082.s006.tiff]
