# Supplementary material for: Scalable and flexible inference framework for stochastic dynamic single-cell models
Source: PLoS Comput Biol. 2022 May 19;18(5):e1010082. doi: 10.1371/journal.pcbi.1010082 (PMC9159578; doi:10.1371/journal.pcbi.1010082)
Supplement: Attachment — Submitted filename: report.pdf [file pcbi.1010082.s008.pdf]

Scalable and flexible inference framework for stochastic dynamics single-cell models

Persson *et al.*

In this work, the authors present a comprehensive computational package for inference of stochastic models that include both intrinsic and extrinsic noise, as well as measurement error. The authors showcase their software tool using a number of synthetic examples, as well as a model of carbon source utilisation in budding yeast. Overall this is a significant and piece of work. However, I have a number of comments that should be addressed before the manuscript can be considered further for publication.

1. It is unclear to me what the real contributions, in terms of new methods or advances, are of the manuscript. My understanding is that the authors take existing modelling frameworks and inference tools and implement them using Julia. The extent to which the manuscript contributes novel understanding or methods should be clearly stated.

2. Since this is a manuscript that effectively provides a computational tool for inference of complicated, stochastic models, the authors should make sure to have developed their code using best software-engineering practices, and to have conducted suitable tests to verify that each method is properly implemented. I could not see any details to this effect in the manuscript, however without such tests it is impossible to be confident in any of the results, or the software itself.

3. The authors state that the default is to allow parameters to vary weakly between cells, or to be constant. Where does the need for such a restriction come from, and to what extent is it possible to relax it?

4. In general I find it really difficult to get much from the results section because all the details are hidden either in the methods (or mostly in the Supplementary Information). The authors should consider completely re-writing this section.

5. Application to simulated circadian clock gene expression model – the authors suggest that the inaccuracy in estimating the breakdown rates τ_2 and τ_4 comes from the fact that only 40 cells are simulated. Have they tested this hypothesis by generating synthetic data for additional cells?

6. Application to simulated stochastic bistable model – the authors state that the use of PEPSDI allows for better mechanistic understanding of the model. However, I don't see what insights are gained, over and above those that could have been gained from simple forward simulation of the model before attempting inference?

7. Performance of adaptive MCMC proposals – the authors should be clear on the new advances provided in this section. If it is to provide new sampling schemes then these schemes should be presented in the Results section. If the purpose of the section is, on the other hand, to benchmark a number of sampling schemes then the authors should choose test problems that have tractable likelihoods so that the accuracy of each method can be evaluated in terms of the fidelity of the generated posterior distributions.

8. Following on from this point, it is generally erroneous to assume that the method that provides the best ESS is the most accurate. The ESS does not quantify the quality of the simulated posterior.

9. Finally, in this section, it is (again) impossible to make sense of the results because so few details of the test cases are included.

10. The authors do not discuss the issues of identifiability and model misspecification at any point. However, it seems to me that both are issues in at least one of the examples used in the manuscript. For example, in the Mig1 study, the authors highlight that there is correlation between c_1 and c_2 but they do not show any bivariate posteriors so the severity of this issue is hard to diagnose.

11. The authors state several times that their method is designed for single cell fluorescence data, however they do not provide any details of how quantitative measurements are obtained from their imaging studies, nor do they discuss the relevance of their noise model (Gaussian, fixed variance) for the specific data that they use.
