# Supplementary material for: Scalable and flexible inference framework for stochastic dynamic single-cell models
Source: PLoS Comput Biol. 2022 May 19;18(5):e1010082. doi: 10.1371/journal.pcbi.1010082 (PMC9159578; doi:10.1371/journal.pcbi.1010082)
Supplement: Attachment — Submitted filename: Rebuttal_pepsdi.pdf [file pcbi.1010082.s009.pdf]

Rebuttal Scalable and flexible inference framework for stochastic dynamic single-cell models

Reviewer 1

In this work, the authors present a comprehensive computational package for inference of stochastic models that include both intrinsic and extrinsic noise, as well as measurement error. The authors showcase their software tool using a number of synthetic examples, as well as a model of carbon source utilisation in budding yeast. Overall this is a significant and piece of work. However, I have a number of comments that should be addressed before the manuscript can be considered further for publication.
We would like to thank the reviewer for a very careful and thorough evaluation of the manuscript. We have received very much appreciated, constructive and valuable comments and suggestions. We carefully addressed all comments which lead to a revision of the manuscript and the supplementary information.

1. It is unclear to me what the real contributions, in terms of new methods or advances, are of the manuscript. My understanding is that the authors take existing modelling frameworks and inference tools and implement them using Julia. The extent to which the manuscript contributes novel understanding or methods should be clearly stated.
   We agree with this comment and apologise for not making this clearer. In the result section paragraph section (ln 134-153), and in the first paragraph of the discussion (ln 323-328), we now elaborate on what is novel, and which parts glues together existing tools in our inference framework.

2. Since this is a manuscript that effectively provides a computational tool for inference of complicated, stochastic models, the authors should make sure to have developed their code using best software-engineering practices, and to have conducted suitable tests to verify that each method is properly implemented. I could not see any details to this effect in the manuscript, however without such tests it is impossible to be confident in any of the results, or the software itself.
   As discussed in Supplementary Note S5.4 we have tested our inference framework by comparing it against the results produced for the Ornstein-Uhlenbeck (OU) model. This model provides a suitable test case since we can exactly compute the likelihood function via the Kalman filter. We therefore compared our inference results against the results obtained when Bayesian inference uses the exact likelihood. Besides testing the Gibbs-sampler for the OU models, we have also tested the other core components, namely the stochastic simulators in our framework, by comparing them against the solvers in the DifferentialEquations.jl package in Julia. We have now added details about the OU model, and the tests of the stochastic simulators in the method section of the manuscript (ln 519-526). We have also made changes in the GitHub project to make the tests of the stochastic solver clearer. Further, in this paper we do not introduce (or claim) to introduce a full package/software (making PEPSDI a full package is future work). To be able to test the performance of our algorithm we tried to make the code as (re)usable as possible.

3. The authors state that the default is to allow parameters to vary weakly between cells, or to be constant. Where does the need for such a restriction come from, and to what extent is it possible to relax it?
   In paragraph three of the first result section we state that letting parameters vary between cells is the default option, but we have rewritten to make this clearer (ln 133-143). We never state that the user is restricted to either option. Rather, in the methods section and extensively in the Supplementary material (S2.4, S2.5.2, S.3, Fig. S5) we provide both the benefits and potential drawbacks of using either option. As stated in the results section, we selected the approach having weakly varying parameters as default because it substantially speeds up inference while still returning precise inference.

4. In general I find it really difficult to get much from the results section because all the details are hidden either in the methods (or mostly in the Supplementary Information). The authors should consider completely re-writing this section.
Our aim with the result section is to keep it accessible for a broad audience. This includes experimentalists in order to make them aware of how modelling and our framework can help with interpreting single-cell data. For those interested in the mathematical details behind the framework, we put substantial effort into writing the supplementary material. We know that this, unfortunately, leads to a loss of mathematical details in the result section, and that in our manuscript we have not been clear enough of where we provide a detailed mathematical description. We have now made this more explicit in the manuscript where the mathematical descriptions can be found (ln 152-153). For each simulation example we have also made more explicit where all simulation/inference details can be found (ln 164 and ln 189-190).

5. Application to simulated stochastic bistable model – the authors state that the use of PEPSDI allows for better mechanistic understanding of the model. However, I don't see what insights are gained, over and above those that could have been gained from simple forward simulation of the model before attempting inference?
Simple "forward simulation" implies that that researcher has manually imputed ad-hoc values of the parameters into the computer simulator, following some a-priori knowledge (in the best scenario) or otherwise imputing rather arbitrary parameter values. This would result into a trial-and-error series of attempts to produce simulated data that could appear similar to real data "by eye". However in this way the choice of the parameters would be not principled, because unconditional to actually available data. Moreover, this approach would not offer the researcher any measure of variability about the parameter values that are more probable (for the given data), that is pure forward simulation would give no uncertainty quantification about the parameters. On the other hand, for example, Figure 3 tells us that μ has most probable values between 7.19 and 7.24, for the data at hand; log(c1) has most probable values between -2.20 and -2.16, and so on. These are values that we do not need to guess via forward-simulation, because our approach can return them to us.

6. Application to simulated circadian clock gene expression model – the authors suggest that the inaccuracy in estimating the breakdown rates 2 and 4 comes from the fact that only 40 cells are simulated. Have they tested this hypothesis by generating synthetic data for additional cells?
We thank the reviewer for the suggestion, and we have now tested this by simulating 100 cells. Overall with such a number of cells, the bias still persist however the posterior modes are a little closer to ground truth (compared to using only 40 cells), see figure S1(c), and the credibility intervals shrink, suggesting that by increasing the number of cells we are moving closer to the truth. This shows that inferring the variability in these rates is challenging (and much larger data may be needed). We have now included this in the manuscript (ln 170-176).

7. Performance of adaptive MCMC proposals – the authors should be clear on the new advances provided in this section. If it is to provide new sampling schemes then these schemes should be presented in the Results section. If the purpose of the section is, on the other hand, to benchmark a number of sampling schemes then the authors should choose test problems that have tractable likelihoods so that the accuracy of each method can be evaluated in terms of the fidelity of the generated posterior distributions.
The aim was to compare three existing adaptive MCMC schemes, which were developed by the respective authors for models having exact likelihoods, in the setting of pseudo-marginal inference where instead a stochastic approximation to the likelihood is used. To make this clearer we have reformulated the first paragraph (ln 201-208). The second model we performed the benchmark for, the Ornstein-Uhlenbeck (OU) state-space model, has a likelihood that can be computed exactly using the Kalman filter. Hence its posterior is available "exactly" (up to the Monte Carlo error induced by running a finite number of MCMC iterations). Using this fact, we ran each adaptive MCMC scheme and used posterior draws to approximate the Wasserstein distance between each posterior resulting from pseudo-

marginal methodology against the gold-standard exact posterior (see ln 219-228 and Fig 4). We approximated the distance using the last 15,000 samples of the exact and pseudo-marginal posteriors (due to computational limitations using more than 15,000 samples is not feasible). The reason why we also benchmarked the MCMC proposal samplers for a model without a tractable likelihood (the Schlögl model) is that, as stated in the text (ln 210-212), this is an interesting case study because the estimated likelihood has a large variance, that is, we are far away from the setting these samplers were developed for.

8. Following on from this point, it is generally erroneous to assume that the method that provides the best ESS is the most accurate. The ESS does not quantify the quality of the simulated posterior.

   The reviewer is absolutely right. We have now added the following analysis in lines 218-228 and Figure 4: since the OU model admits an exact likelihood (via the Kalman filter) we compare the PEPSDI-provided posterior with the gold-standard posterior (where the exact likelihood is used) by computing the Wasserstein distance between the posteriors (or, more precisely, by approximating it using posterior samples). The comparison is, once again, favourable to the RAM sampler which displays the smallest distance and also the smaller variability, and hence robustness to varying number of particles and starting parameter values.

9. Finally, in this section, it is (again) impossible to make sense of the results because so few details of the test cases are included.

   With the same motivation to point 4, we kept the main text brief. We have provided additional details in the caption for Fig. 4. We agree that the amount of details regarding the benchmark is sparse. Hence, in the supplementary we have added a detailed description of the benchmark setup (section S5.5). It should be noted that we do not aim to a comprehensive benchmark in this paper, rather, we think it might be interesting for future papers to know about the results we got. To emphasise this, in the discussion we have added that future benchmarks might also consider different tuning parameters for the adaptive MCMC schemes (ln 375-377).

10. The authors do not discuss the issues of identifiability and model misspecification at any point. However, it seems to me that both are issues in at least one of the examples used in the manuscript. For example, in the Mig1 study, the authors highlight that there is correlation between c_1 and c_2 but they do not show any bivariate posteriors so the severity of this issue is hard to diagnose.
    Regarding identifiability: we apologise for not addressing the issue of identifiability for the Mig1 model (which is especially relevant as the data is not simulated). Unlike the case for deterministic mixed-effects ODE models there is (to our knowledge) a lack of computationally efficient rigorously established methods (like profile likelihood) for identifiability for mixed-effects models with (tau-leaping) stochastic models. Developing such a method is relevant, but unfortunately outside the scope of this paper. Still, to address this issue we used the relatively simple approach of launching 8 MCMC runs from different (randomly sampled from the prior) starting locations and inspecting whether they converged to the same region. Overall, we found that there appears to exist different modes since for some parameters the chains converge to different areas (Supplementary Fig. S4), suggesting that the model is not identifiable in the sense that multiple parameter values provide the same model fit. One should be careful about interpreting the parameters as we do in Fig. 6h when we cannot determine unique best fitting parameters, however, we noticed that for each inference run (and even though we had convergence to different modes) the relationship we present in Fig. 6h hold, that is (i) (c_1, c_2) are inferred to be strongly correlated, (ii) the coefficient of variation in (c_1, c_2) is larger with decreasing fructose and (iii) μ_2 is larger in weak fructose. As these properties hold for various inference runs, this strongly suggests that in order for the model to fit the data these properties most hold. Overall, we have revised the text to highlight that our model does not appear to be fully identifiable (hence the absolute values should be carefully interpreted), but that certain parameter relationships always appear to hold (ln 278-286, Fig. S4).

Regarding correlation, we are careful to call correlation between parameters an issue, as correlation (or co-regulation of biological processes) is an often occurring important biological property for single-cell dynamic models. That is why we use a population parameter distribution, $\pi(\mathbf{c}^{(i)}|\boldsymbol{\eta}$, where we can infer for $(c_1, c_2, Mig1c_{t_0}, Mig1n_{t0})$, besides the log-mean and variance, their correlation matrix $\boldsymbol{\Omega}$ (see Fig. 6c). To make this cleaerer We have also emphasised that what we show in the right part of Fig. 6h is the posterior of $\boldsymbol{\Omega}_{1,2} = corr(c_1, c_2)$. We agree that having correlation can cause problems with the inference, and we thank the reviewer for suggesting pair plots. Overall, in the pair plots for the correlated features (Fig. S5) we can notice correlation (for example in the log-mean of (c_1, c_2)) but also that it does not seem to severely hamper the inference of the posterior (Fig. S4) (we do not provide additional pair plots for all inferred parameters as that would result in > 40 plots).

Regarding misspecification, we agree that all models in biology are likely to be misspecified due to the complexity of biological systems. However, as the SNF1 pathway (of which Mig1 is a part) is extensively studied – and we have substantial experience in working with this pathway – we do not think model misspecification plays a large role here. As we also state in the text on the assumptions behind the model (ln 248-259), if new knowledge of the SNF1 pathway overthrows our model assumptions, we believe the reader will be able to identify any, potential, incorrect model assumption.

11. The authors state several times that their method is designed for single cell fluorescence data, however they do not provide any details of how quantitative measurements are obtained from their imaging studies, nor do they discuss the relevance of their noise model (Gaussian, fixed variance) for the specific data that they use.
We have now included further details on the experimental procedure, please see ln 506-548. Regarding Gaussian measurement noise, we followed previous publications working with similar data in the context of the SNF1 pathway that had found this measurement noise to work well. We have now clarified this in the Methods section (ln 527-568), and in the supplementary.

Reviewer 2

In this article, the authors describe PEPSDI (Particles Engine for Population Stochastic DynamIcs) a modelling framework that infers model parameters from dynamic data for single-cell models that account for both intrinsic and extrinsic noise. Their framework is a Bayesian inference framework with a new inference method capable of modeling intrinsic noise with different simulators. The authors argue that this work is important due to the need to extract mechanistic information from single-cell dynamic data (e.g. single-cell microscopy) in particular to better describe and understand cell-to-cell variability. While there are existing inference methods, they suffer drawbacks (ignore noise, assume time invariant constants, other unrealistic model assumptions) that the flexibility in this framework overcome. The authors apply their framework to simulated biological systems (circadian clock, Schlogl bistable toy model) as well as to time-lapse microscopy data of the Mig1 transcription factor to identify drivers of heterogeneity in its localization under different nutrient conditions. As strength of the article is that the code is freely available, and perhaps more importantly for adoption, well documented with tutorial examples. Overall, the authors are working on an important problem, namely, how can we understand intrinsic and extrinsic noise in biological systems and infer mechanistic model parameters? Their method should be adoptable and implementable for other important biological systems, outside of yeast and the toy simulated models detailed here. I have a few comments that should be addressed prior to publication:

We would like to thank the reviewer for a very careful and thorough evaluation of the manuscript. We have received very much appreciated, constructive and valuable comments and suggestions. We carefully addressed all comments which lead to a revision of the manuscript and the supplementary information.

1. Why were known sources of extrinsic noise not incorporated into the Mig1/SNF1 model? For example, cell cycle, cell age, and the metabolic cycle are all know to impinge on the relevant

metabolic processes.

Extrinsic noise factors such as the cell cycle state, and lineage effects are included in the model. Specifically, in the model we incorporate and lump these noise sources by letting the "uppstream" rates (c_1, c_2), vary between cells. Moreover, since extrinsic noise is often slow [1], and we have experimental data for 15 minutes, we assumed as in previous studies employing single-cell dynamic modelling that these noise sources should materialise in stable differences between cells in the form of constant rate constants. Overall, the reason why we lump a lot of sources into (c_1, c_2) is because we cannot disentangle between effects from, for example, the cell cycle and lineage with our experimental setup. Besides this uppstream extrinsic noise, to account for cell varying protein numbers, we let the initial Mig1 values vary between cells. We realise we are not clear about how noise enters into the model in the text, and also following reviewer 3 we have now revised the text to make this clearer (ln 260-269).

2. How much data was collected for the Mig1 experiment (n=?) I may simply be overlooking the number, but it seems like a relatively small number of cells. Particularly given the ease of collecting localisation data from hundreds of cells. More generally, is there a justification for the numbers of cells used? For example, 40 simulated cells were used for the circadian clock model.

   In both experiments the amount of individuals in the experiment is mentioned in the figure caption. We realise that this was not clear enough, so we have rewritten this part (see figure caption for Fig 5.). The reviewer makes a valid point that the number of cells is relatively small in the first experiment. However, in this case we look at the early reaction of Mig1, which is very fast ($<$ 30 sec). Therefore, we need a microfluidics device which has a strict spatial and temporal control of the conditions the cells are exposed to. The microfluidics system used in this work gives a switching time of 300 ms, this is the time it takes for all the cells in the sample to be stably exposed to the exact concentration. Meaning that we only introduce a 300 ms experimental variability in our measurements between individuals. However, this comes with a disadvantage which is a restriction in the area that is exposed (± 40x90 µm), this severely reduces the amount of yeast cells that can be exposed for the conditions upshift. Microfluidics device can capture and observe a large number of cells. Previously we have also done this [2]. The second set of experiments is done with this device and captured +/- 130 cells, however this larger number of cells comes at a loss of precision when the cells where exposed to fructose.
   For the circadian clock model we used 40 cells to show that our framework can work when the number of cells is few. But, as we show in Fig. S3 the framework scales quite well with the number of individuals highlighting that handling bigger data sets is possible. To make it clearer that our framework can handle more than 40 cells, we now have changed the number of cells used for the Schlögl model to 150 cells (Fig. 3).

3. Please clarify your justification of using the Extrande algorithm.
   We used the Extrande simulator since it can handle time-varying kinetic parameters (in this case the transcription rate), which the SSA simulator cannot. This has now been clarified (ln 165-168).

4. Some additional details are needed in the methods describing the Mig1 experiments. Please include strain construction details or source. Also please include additional details on how Mig1 nuclear localization was quantified. Was the nuclear marker used to identify the nucleus? How were cells segmented? How the quantitative measurement are done is currently omitted from the manuscript.

   We apologise for omitting these crucial experimental details. We have now included additional explanation of the experimental setup in the Materials and Methods (ln 506-548). Shortly, the nuclear marker was not used for the identification of the nucleus. Instead the 15% brightest pixels where taken and the nuclear marker was taken as a control if this was correct. The CellX software was used for the segmentation and quantification of the experimental data.

5. I do not see where the Hxk1 expressing plasmids are detailed in the Methods/supplementary material. Was this overexpression of Hxk1?
   The strain source and construction details of the Hxk1 expressing strain are described in the paper [2]. The reviewer is correct in that this is not clearly written in the MM and we have clarified that the strain is taken from this paper which also contains the information on the construction of the plasmid. This plasmid expresses HXK1 from a promotor that is deregulated from nutrient conditions and it this plasmid should express Hxk1 in higher amount then the native promotor. We have shown that this plasmid restores the glucose phosphorylating activity to levels higher than in the wild type. See the text ln 506-548.

Reviewer 3

This is a very useful piece of work. I found the statistical analysis well explained and laid out. Distinguishing between extrinsic and intrinsic noise is challenging and important and I believe that this is a timely and useful contribution.

I have two very minor points that I would like the authors to consider, that I want to mention first: - I would have liked to see the discussion of PEPSDI moved up at the front of the results. I found the SI very useful and detailed; and I have spend a lot of time on this material. I regard this as outstanding and very clear. - I think that the discussion of extrinsic noise could be sharpened up and there are references that could be usefully added to give a comprehensive picture of the role of extrinsic noise and how we can identify it's contribution to cell to cell variability.

We would like to thank the reviewer for a very careful and thorough evaluation of the manuscript. We have received very much appreciated, constructive and valuable comments and suggestions. We carefully addressed all comments which lead to a revision of the manuscript and the supplementary information.

1. pg 3, 2nd paragraph: [13] is an odd choice to dismiss alternatives to Extrande. It is too generic in my view even though it applies generally. I think Schnoerr et al (doi:10.1088/1751-8121/aa54d9) is more suitable in this context.

   Thank you for pointing us to this reference. We agree that this is more suitable and we have now included it in the ms.

2. pg 3, 8th paragraph: how was runtime for PEPSDI determined?

   When determining the run-time for the Schlögl model in Fig. S3 we used the wall-clock time (not cpu time). This has now been added in the main text (ln 141-142). Furthermore, the benchmark was done on a freshly rebooted laptop to avoid noise from other programs, and each run was performed sequentially using a single core. We further performed inference on the Langevin approximation of the Schlögl model using a guided particle filter, overall we used the same setup as for the example in the paper. These details have now been included in the caption for Fig. S3. The fact that the runtime is highly consistent between runs (see the tiny error-bars in Fig. S3) shows that possible computational noise during the runs had a negligible effect on the timing results.

3. Fig 2: I would like to learn more about the joint densities over inferred parameters. In my work I have found e.g. all pairwise plots useful and especially for tau2-tau4 I would like to see such plots (also in relation to the mu parameters). Similar for Figure S1.

   Thank you for this great suggestion. We have now included for the clock model in Fig. S1, for the Schlögl model in Fig. S2 and for the Mig1 model in Fig. S4 relevant pair plots. With relevant we mean the pair plots between correlated variables, or those that were problematic to infer, like τ_2 for the clock model. Unfortunately we could not include all possible pair plots in the manuscript, since for example in the clock model there are 8 choose 2 = 28 possible pair plots (but they are included in the github repository).

4. Fig 3 (and corresponding section on pg3,4): what were the parameters chosen for the MCMC scheme chosen here? S3 contains some detail, but are 10,000 iterations enough?

We believe the reviewer here refers to Fig. 4 (benchmark of adaptive MCMC), as in Fig. 3 we used 500,000 iterations (now 100,000 when we have increased the number of cells addressing reviewer 2). We apologise for not including more details for benchmarking the adaptive MCMC schemes. In Supplementary Note S5.5 we now provide full details of the model setup (parameters used, priors, number of particles for pilot run), and also the options we used for the adaptive MCMC-schemes. We also have added in the discussion that our results might depend on how we tuned the adaptive MCMC samplers (ln 375-377). We also do not use 10,000 iterations (ln 213), but 60,000 for each run in the benchmark. This has now been clarified. We have further rewritten parts of the adaptive MCMC section to make everything more clear.
If 10,000 iterations refer to measuring the run-time of the Schlögl model in Fig. S3, we believe it is enough to get a good idea of the difference in run time since the variability between different runs (error bars) is very small. Furthermore, we are doing the inference using the SDE approximation (which is now more clear in text) of the reaction system, so we do not expect the runtime to vary substantially due to the underlying stochastic simulator.

5. pg 4-5: the examples are outlined in welcome brevity, but I would have liked a clearer discussion of where extrinsic noise enters (see e.g. the discussion in Filippi et al (doi: 10.1016/j.celrep.2016.05.024)

   Thank you for pointing this out, and we agree that we should make this more clear. In the section *Modelling of Mig1 nuclear dynamics* we have now made more explicit how we model the two sources of extrinsic noise we consider. Specifically 1) the extrinsic noise sources arising upstream of the SNF1 pathway which is related to metabolic activity and 2) the extrinsic noise arising from varying protein levels within the Mig1 nuclear shuttling pathway (ln 260-269). Furthermore, in the section *Cell-to-cell variability in Mig1 nuclear dynamics is tightly regulated by fructose availability* we have now included an additional inference results, showing that the model is not capable of describing the data without the upstream extrinsic noise (ln 289-291). This highlights how uppstream extrinsic noise appears to be a major contributor of cell-to-cell variability in the observed Mig1 shuttling dynamics.

6. pg 6-7 discussion: The discussion here struck me as slightly superficial. I think the authors' contribution is not done sufficient justice. A clearer discussion of the roles of extrinsic noise could help. It is fiendishly difficult to measure extrinsic noise experimentally and distinguish it from intrinsic noise(Fu Pachter, doi: 10.1515/sagmb-2016-0002). Dual reporter assays (Hilfinger and Paulsson 10.1073/pnas.1018832108), are difficult to implement; and while other methods are becoming available (Ham et al doi: 10.7554/eLife.69324; Gorin and Pachter doi: 10.1101/2020.09.25.312868) statistical inference of models such as performed here will be our best bet to get a handle on extrinsic noise.
   Thank you for pointing this out, we fully agree. We have now included a new paragraph in the discussion where we discuss with more detail on how modelling via a framework such as ours can disentangle noise sources (ln 349-358). Furthermore we have now included a brief discussion on the importance of developing better particle filters (ln 362-368), and some additional details on our benchmarking of adaptive MCMC (375-377).

References

[1] Loos C, Hasenauer J. Mathematical modeling of variability in intracellular signaling. Current Opinion in Systems Biology. 2019 aug;16:17-24.

[2] Schmidt GW, Welkenhuysen N, Ye T, Cvijovic M, Hohmann S. Mig1 localization exhibits biphasic behavior which is controlled by both metabolic and regulatory roles of the sugar kinases. Molecular Genetics and Genomics. 2020 nov;295(6):1489-500.
